# From metric to model: a five-stage pipeline for building and sustaining valid AI predictive systems in consumer neuroscience

**DOI:** 10.3389/fnhum.2026.1837923

**Published:** 2026-09-29

**Authors:** Thomas Zoëga Ramsøy

**Affiliations:** 1 Neurons, Taastrup, Denmark; 2 International Center for Applied Neuroscience, Rørvig, Denmark; 3 Free University of Languages and Communication (IULM), Milan, Italy

**Keywords:** AI in advertising, consumer neuroscience, machine learning, model governance, neuromarketing, neurometric reliability, predictive validity, Psychometric Validation

## Abstract

The application of machine learning to consumer neuroscience has made it possible, in principle, to predict consumer responses from media assets alone. However, the pursuit of model sophistication has outpaced attention to a foundational constraint: the quality of any predictive artificial intelligence (AI) system is bounded by the quality of the neurophysiological and biometric data used to train it, and by the rigor of the modeling procedures applied to those data. This article argues that persistent problems of low metric reliability, poor cross-metric consistency, and the broader replication crisis have left the field building predictive systems on an insecure measurement foundation. Anchored in the framework for neurometric validation established by Ramsøy (2019), the paper offers a conceptual synthesis and agenda-setting framework for the AI era: a five-stage pipeline comprising (1) Metric Mapping, the theory-driven identification and pre-screening of candidate neurophysiological and biometric measures; (2) Psychometric Validation, the formal assessment of each metric’s sensitivity, specificity, validity, and reliability; (3) Discriminatory Ability, an assessment of whether a metric produces meaningful score distributions across real-world commercial stimuli; (4) Principled Data Scaling and Model Development, covering both the large-scale data collection and the modeling and validation procedures required for robust prediction; and (5) Model Governance and Drift Prevention, the ongoing monitoring, retraining, and calibration required to maintain predictive validity over time. The individual components draw on established practice in psychometrics, measurement science, and applied machine learning; the contribution lies in integrating them into a coherent, domain-specific architecture for consumer neuroscience, and in making explicit a criterion—Discriminatory Ability—that is widely assumed but rarely tested in applied advertising research. The pipeline is offered not as a validated standard but as a structured research agenda and a basis for future work on standardization, certification, and governance, with the aim of shifting the field from a model-centric toward a validation-centric culture.

## 1 Introduction: the three steps of predictability

The promise of predicting consumer behavior from brain and biometric data has evolved through three distinct and cumulative stages, each building on the last to bring the field to its current position at the frontier of artificial intelligence–driven prediction. Understanding this progression is essential not merely as historical context, but because the methodological differences between these stages carry profound implications for how AI systems in consumer neuroscience must be approached, built, and evaluated.

The first stage established that neural signals could predict an individual’s imminent behavioral choices. Foundational work by Libet and colleagues showed that electrical brain activity measurably precedes conscious awareness of a decision (Libet et al., 1983). The principle was extended to economic choices: functional magnetic resonance imaging showed that activity in the nucleus accumbens, associated with outcome anticipation, could predict a purchase moments before the person consciously committed to it (Knutson et al., 2007), demonstrating that the brain encodes value in ways legible to an external observer. Later work extended this to a more naturalistic context, showing that prefrontal EEG asymmetry could predict willingness-to-pay. That study is instructive here: it assessed the alpha, beta, and gamma bands and found only gamma-band asymmetry significantly related to willingness-to-pay, with a trend in beta, while alpha-band asymmetry—the index conventionally reported and assumed to index approach motivation—showed no significant relationship (Ramsøy et al., 2018). A metric’s conventional standing is therefore not evidence of its validity for a particular task. That study was small and used consumer-grade recording, and its findings await replication; the point is not the result’s strength but the pattern it illustrates. These studies were concerned with the predictability of individual behavior from individual neural data.

The second stage scaled this principle from individuals to markets, giving rise to neuroforecasting. This work revealed a striking finding: the neural responses of as few as thirty to fifty participants could predict in-market success at the population level, sometimes months or years in advance. Landmark studies forecast movie box office revenue (Berns and Moore, 2012), the commercial success of film trailers (Boksem and Smidts, 2015), and the degree to which online content would be shared (Falk et al., 2012) or engaged with at population level (Dmochowski et al., 2014). These findings mattered because they suggested that neural responses are not merely idiosyncratic but carry a shared, population-level signal about what will resonate, engage, and persuade (see Genevsky and Yoon, 2022, for an overview).

It matters to characterize what neuroforecasting studies did and did not do. Neurophysiological data were collected from participants and analyzed statistically—typically as correlations or linear regressions between group-average responses and subsequent market outcomes—to establish a predictive relationship. They were not studies in which models were trained to make autonomous predictions about new stimuli without collecting additional human data. The distinction is fundamental: neuroforecasting demonstrated that neural signals contain recoverable information about future market success; it did not create a system capable of extracting that information from new creative content without ever measuring a brain.

The field has recently entered the third and most ambitious stage: building AI models that predict consumer responses from media assets alone. By training machine learning algorithms on large datasets of neurophysiological and biometric responses to thousands of stimuli, the goal is to create models that no longer require data collection for each new prediction. A practitioner could, in principle, upload a new advertisement and receive an instant, AI-generated prediction of its likely attention score, emotional engagement profile, and memory encoding potential—without a single participant ever viewing it. This represents the ultimate scaling of consumer neuroscience: a democratization of the insights that previously required expensive and time-consuming laboratory studies.

However, the transition from the statistical correlations of neuroforecasting to the predictive automation of AI is not a simple incremental step; it is a methodological leap that demands a fundamentally different level of rigor regarding input data quality. In neuroforecasting, the researcher collects the data, controls experimental conditions, and interprets the resulting statistical relationships. In AI-driven prediction, the algorithm must learn a generalizable function from training data and apply it blindly to new, unseen inputs. This places an enormous burden on the quality, consistency, and representativeness of the training data. A model can only learn what its data teaches it. If the data is noisy, inconsistent, or based on metrics that are not valid measures of the underlying psychological constructs, the model will learn noise, inconsistency, and invalidity.

This is where the field has fallen critically short. The foundational work of building valid and reliable neurometrics—a prerequisite for any sound predictive system—has been dangerously neglected in the rush to develop AI models. As established in earlier foundational work (Ramsøy, 2019), any metric used in this field must meet criteria of sensitivity, specificity, validity, and reliability. Without this, the field risks building its AI infrastructure on an unstable and unvalidated foundation.

This concern is not confined to consumer neuroscience. It converges with a broader movement toward AI governance, transparency, and risk management in applied commercial systems, reflected in regulatory instruments such as the European Union’s Artificial Intelligence Act (European Parliament and Council of the European Union, 2024) and in emerging documentation norms such as model cards (Mitchell et al., 2019). These frameworks share a common premise: systems that inform consequential decisions should be accompanied by evidence about how they were built, what they were trained on, and how their performance is maintained over time. The argument developed here is a domain-specific contribution to that wider effort.

Its urgency is heightened by the rapid adoption of generative AI in advertising creative production and testing. When creative variants can be generated in minutes and at volume, the pressure to evaluate them through automated prediction rather than human data collection increases sharply. This makes the validity of the underlying metrics, and the soundness of the models trained on them, more consequential rather than less.

The present paper therefore offers a conceptual synthesis and agenda-setting framework: a five-stage pipeline for building and sustaining valid predictive AI systems in consumer neuroscience. Its components are drawn from established practice in psychometrics, measurement science, and applied machine learning; the contribution lies in their integration into a coherent architecture adapted to the specific conditions of this domain, and in identifying where current practice falls short at each stage. It is offered primarily as a research agenda for academic investigators, and secondarily as a set of considerations relevant to those who develop and evaluate applied predictive systems.

## 2 The foundational problem: a crisis of measurement

Before detailing the proposed pipeline, it is necessary to understand the depth and breadth of the measurement crisis that motivates it. The path from academic discovery to a scalable, reliable metric suitable for AI training is fraught with obstacles. Three core and deeply interconnected problems stand in the way: the replication crisis in the underlying science, the endemic inconsistency of neuromarketing metrics, and the inherent complexity of biostatistical data.

### 2.1 The replication crisis and its implications for AI training data

Neuroscience and psychology are in the midst of a well-documented replication crisis. The Open Science Collaboration’s landmark study, which attempted to replicate 100 published psychological experiments, found that only approximately 36% of replications yielded statistically significant results, and the average effect size of replications was approximately half that of the original studies (Open Science Collaboration, 2015). This is a consequential finding for a field that aspires to build AI models from accumulated scientific literature. If the published effect sizes are systematically inflated—a consequence of academic incentive structures that reward novel, positive findings over null results and replication studies—then any metric shown to be predictive in a single, small-scale study is a weak and potentially unreliable candidate for building a robust AI model.

The mechanisms are well understood. Pressure to publish significant results encourages practices that, while not fraudulent, capitalize on chance: selective outcome reporting, hypothesizing after results are known (HARKing), and flexible analytical choices applied until significance is reached (Aguinis et al., 2017). The result is a literature in which a metric-outcome relationship may rest on a single underpowered study with an inflated effect size that would not replicate elsewhere. The implication for AI is direct: machine learning models learn spurious patterns as readily as real ones. Trained on metrics whose validity rests on a single unreplicated study, a model learns to predict that metric’s behavior, not the construct it purports to represent—technically valid, in that it predicts the training labels, but scientifically invalid, because those labels are not reliable measures of the construct they claim.

Furthermore, academic studies in consumer neuroscience are primarily designed to test hypotheses within controlled experimental settings, not to create metrics that are consistent and reliable across diverse studies, contexts, and populations. Study designs vary considerably between laboratories in terms of stimuli, participant populations, data acquisition equipment, preprocessing pipelines, and the specific operationalization of the construct of interest. This variation, which is natural and even desirable in scientific exploration, is deeply problematic for building large-scale, consistent training datasets. Science is driven by systematic variation on a theme; AI training requires consistency of a theme.

### 2.2 The inconsistency of neuromarketing metrics

The second foundational problem is the profound lack of consistency within the commercial neuromarketing landscape. This problem was brought into sharp relief by a landmark study by Varan et al. (2015), which compared measures of advertising effectiveness produced by multiple commercial neuromarketing vendors when all were exposed to the same set of advertising stimuli. The findings were striking and troubling: vendors’ measures of the same psychological construct—most notably “engagement,” the single most widely used metric in applied neuromarketing—showed little to no correlation with one another.

This constitutes a direct, empirical demonstration of the failure of parallel-form validity. In classical psychometrics, parallel-form validity (sometimes called alternate-form reliability) refers to the degree to which two different operationalizations of a test designed to measure the same construct produce consistent results. If two thermometers placed in the same glass of water give different readings, at least one is broken. The findings of Varan et al. (2015) suggest that the field’s thermometers collectively give wildly different readings of the same phenomenon. This is not merely an academic concern; it is a fundamental problem for the credibility of the entire enterprise of AI-driven consumer neuroscience.

The root cause of this inconsistency is a field that has prioritized proprietary differentiation over the transparent, collaborative work of establishing shared, validated constructs. Each vendor has developed its own proprietary algorithm for computing “engagement,” “attention,” or “emotional response,” often without publishing the algorithm’s details or subjecting it to independent validation. The result is a marketplace of metrics that are, in a very real sense, incommensurable. They cannot be meaningfully compared, aggregated, or used as the basis for a shared scientific understanding.

The implications for AI are again direct. A model trained on one vendor’s measure of “engagement” is fundamentally different from one trained on another’s measure of the same construct; if the two measures are uncorrelated, the two models are effectively predicting different phenomena even though both are marketed identically. In an ideal state, metrics purporting to measure the same construct should show high inter-metric correlation, indicating convergence on the same underlying reality. The available evidence suggests they do not. More recently, reliability analyses of EEG-based advertising metrics have begun to document this problem more precisely, highlighting the critical gap between the field’s implicit reliability assumptions and its empirically documented reliability levels (Van Diepen et al., 2024).

### 2.3 The inherent complexity of biostatistical data

The third foundational problem is the inherent nature of the data itself. Neurophysiological and biometric signals are not simple, clean, tabular data of the kind that standard machine learning algorithms were designed to process. They are complex, high-dimensional, non-stationary time series subject to immense individual variability, susceptible to numerous sources of noise and artifact, and highly sensitive to the conditions under which they are collected. This is precisely why biostatistics exists as a specialized scientific discipline.

EEG signals, for example, are contaminated by muscle artifacts (particularly those generated by eye movements and blinks), environmental electrical interference, and amplifier noise. The preprocessing pipeline required to extract a meaningful signal from raw EEG data involves multiple steps—including filtering, artifact rejection, and independent component analysis—each of which involves methodological choices that can significantly affect the derived metric. Different laboratories employ different preprocessing pipelines, and the resulting metrics can differ substantially even when derived from identical raw data. Similarly, galvanic skin response (GSR) signals are highly sensitive to ambient temperature, electrode placement, and individual differences in baseline skin conductance. Heart rate variability is affected by respiration, movement, and a host of additional physiological factors.

This variability is compounded by the growing heterogeneity of acquisition hardware. Research-grade laboratory systems now coexist with a rapidly expanding market of wearable and portable devices—consumer EEG headsets, webcam-based eye tracking and facial coding, wrist-worn electrodermal and cardiac sensors—which differ in electrode count and placement, sampling rate, signal-to-noise characteristics, and proprietary on-device preprocessing. Such devices substantially extend the scale and ecological reach of data collection, which is a genuine advantage for AI training. They also introduce systematic between-device variance that is easily confounded with the constructs of interest. A training corpus assembled across heterogeneous hardware without explicit cross-device validation risks encoding device signatures rather than psychological states, and cross-device comparability should therefore be treated as an explicit validation requirement rather than an assumption.

Academic studies are typically designed to test specific hypotheses within tightly controlled experimental environments, where sources of variability can be minimized and managed. They are not designed to build scalable data collection and processing pipelines robust to real-world noise and variability. Statistical methods in a single paper are often bespoke to its specific design, making principled data aggregation across studies extremely difficult. Any attempt to build a large-scale AI training dataset by pooling data from multiple academic studies without a deep investment in standardized, validated biostatistical processing risks producing a model that learns the idiosyncrasies of different data collection environments rather than the underlying psychological constructs of interest.

### 2.4 A counterargument: when does prediction require valid measurement?

The argument developed so far requires qualification. It would be overstated to claim that psychometric validity is a strict precondition for all useful prediction. In many applied machine learning contexts, models built on noisy or theoretically unmotivated features achieve strong and commercially valuable performance; recommender systems and click-through prediction succeed without any commitment to construct validity. A neurophysiological signal of uncertain construct status may likewise carry reliable predictive information about market outcomes, and where the sole objective is forecasting accuracy against a well-defined outcome, honestly established on genuinely held-out data, a psychometrically impure proxy can have legitimate value.

The distinction that matters is between prediction and explanation. Difficulties arise when outputs are labeled with, and interpreted as, psychological constructs. A system described as measuring “cognitive load” or “memory encoding” makes an implicit construct claim, and users act on it: creative teams revise executions because an advertisement is said to be cognitively demanding, not because an unnamed feature vector returned a particular value. Once construct language is attached to an output, the burden of construct validity applies regardless of how the model was built.

Two positions follow. Purely predictive systems should be described in purely predictive terms, with the training outcome stated and no psychological interpretation implied. Systems that do make construct claims—which describes most applied neuromarketing AI, and which is the source of much of its commercial appeal—require the validation evidence set out below. It is to this second case that the pipeline is principally addressed, and this defines its boundary conditions.

These three foundational problems—the replication crisis, the parallel-form validity failure, and the inherent complexity of biostatistical data—together with the interpretive tension just described, collectively demand a new approach to building AI in consumer neuroscience. The field cannot simply scrape the academic literature, aggregate vendor metrics, or collect data at scale without first solving the measurement problem. Metrics must be built from the ground up, with the end goal of AI training in mind and a level of rigor commensurate with the stakes of the predictions being made. The following five-stage pipeline provides a principled roadmap for doing so.

## 3 Stage 1: Metric Mapping

The first stage of the proposed pipeline, Metric Mapping, is a crucial and routinely overlooked preparatory step. It involves the systematic identification, pre-screening, and theoretical grounding of candidate neurophysiological and biometric measures before any large-scale data collection or model building commences. The core principle of this stage is that the availability of a physiological signal does not, in itself, constitute evidence of its relevance to a target consumer behavioral outcome. A principled pre-screening process is essential to reduce noise, avoid spurious correlations, and ensure that resulting AI models are built on a foundation of theoretically meaningful data.

The landscape of potential metrics is vast. EEG yields frontal alpha asymmetry (approach-avoidance motivation), theta and alpha power (cognitive load and attention), and event-related potentials (time-locked responses). Eye-tracking yields fixations, saccades, and pupil dilation. Galvanic skin response and electrodermal activity index arousal; heart rate and its variability index cardiovascular reactivity reflecting emotional state, load, and stress. Functional magnetic resonance imaging, though rare in applied settings owing to cost and immobility, provides high-resolution regional measures critical for foundational work. A newer class of AI-derived perceptual metrics—models predicting which elements of an image or video capture attention—must meet the same validation standards as traditional biometric measures.

A structured mapping exercise evaluates each candidate against four questions. First, what is the theoretical grounding? There must be a plausible, mechanistically described pathway linking the measure to the outcome, built through substantive engagement with the neuroscience, psychology, and consumer-research literatures rather than superficial citation; the chain from signal to construct to behavior must be coherent, specific, and stated precisely enough to be empirically testable. Second, what prior published evidence supports predictive value in similar contexts? Given the replication crisis (Section 2.1), a single positive study is insufficient; converging evidence across independent groups is needed. Third, what practical constraints apply to capturing the metric at the scale and ecological validity AI training requires? A measure highly sensitive in the laboratory may not survive real-world noise. Fourth, and often overlooked, is the metric meaningful in the deployment context? A disgust or fear index may be perfectly valid yet, across commercial advertising that deliberately avoids aversive content, show near-zero variance and offer no discriminatory value.

This principled approach to pre-screening stands in contrast to the common practice of “kitchen sink” modeling, in which every available signal is included in a training dataset in the hope that the algorithm will identify what is important. While machine learning algorithms are adept at finding patterns in high-dimensional data, they are equally susceptible to capitalizing on chance correlations, leading to models that are technically accurate predictors of theoretically unvalidated proxies. A model might learn that a particular pattern of eye movements predicts clicks in a dataset of digital advertisements, but without theoretical grounding it is impossible to determine whether this pattern reflects genuine consumer interest or simply a design convention common to the advertisements in the training set. Such a model would fail to generalize to new advertising formats and would provide no scientific insight into why it makes the predictions it does.

To make this screening transparent and reproducible rather than a matter of expert intuition, the mapping exercise should be conducted as a structured review rather than an informal literature scan. The reporting conventions of PRISMA (Page et al., 2021) provide a workable template: pre-specified inclusion and exclusion criteria for candidate metrics, documented search strategies across the relevant literatures, explicit recording of the studies consulted for each metric, and a transparent account of why candidates were retained or rejected. A full systematic review is not warranted in every case, and PRISMA was designed for a different purpose; what is borrowed is its discipline of documentation.

The practical output of Stage 1 should therefore be an auditable record—a metric map—stating, for each candidate, its theoretical rationale, the supporting evidence and an appraisal of its quality, known confounds, acquisition constraints, expected commercial variance, and the decision taken. Applied consistently, this means asking which candidate measures have a published mechanistic rationale linking them to the consumer behaviors of interest—attention, emotional engagement, cognitive demand, and memory encoding—before large-scale data collection is initiated, and documenting the answer in a form that reviewers and collaborators can inspect. This disciplined, theory-driven approach to metric selection is the essential first step in building AI models that are not only predictive but also interpretable and robust to distributional shift.

A caution applies to the growing use of AI tools to conduct the literature scoping this stage depends on. Large language models and automated search tools can accelerate the mapping of candidate metrics, but they inherit, and may amplify, the very publication biases described in Section 2.1: null results, failed replications, and contradictory findings are underrepresented in the training corpora and in the highly cited literature such tools surface most readily. If Stage 1 is delegated to an automated scan without correction, the resulting metric map will over-represent the optimistic published record. Where AI assistance is used, it should therefore be treated as a first pass subject to human verification, with a deliberate, human-led search for disconfirming evidence—registered-report and replication literature, null-result repositories, and adversarial or critical commentary—so that a metric’s apparent support is not simply an artifact of what the underlying tools were most likely to return. The provenance of the evidence supporting each candidate, including whether it was surfaced by automated or manual search, belongs in the metric map.

The criteria illustrated in Figure 1—sensitivity and specificity against a controlled ground truth—represent the minimum a candidate metric must satisfy before it can be considered for the Psychometric Validation of Stage 2.

**Figure 1:**
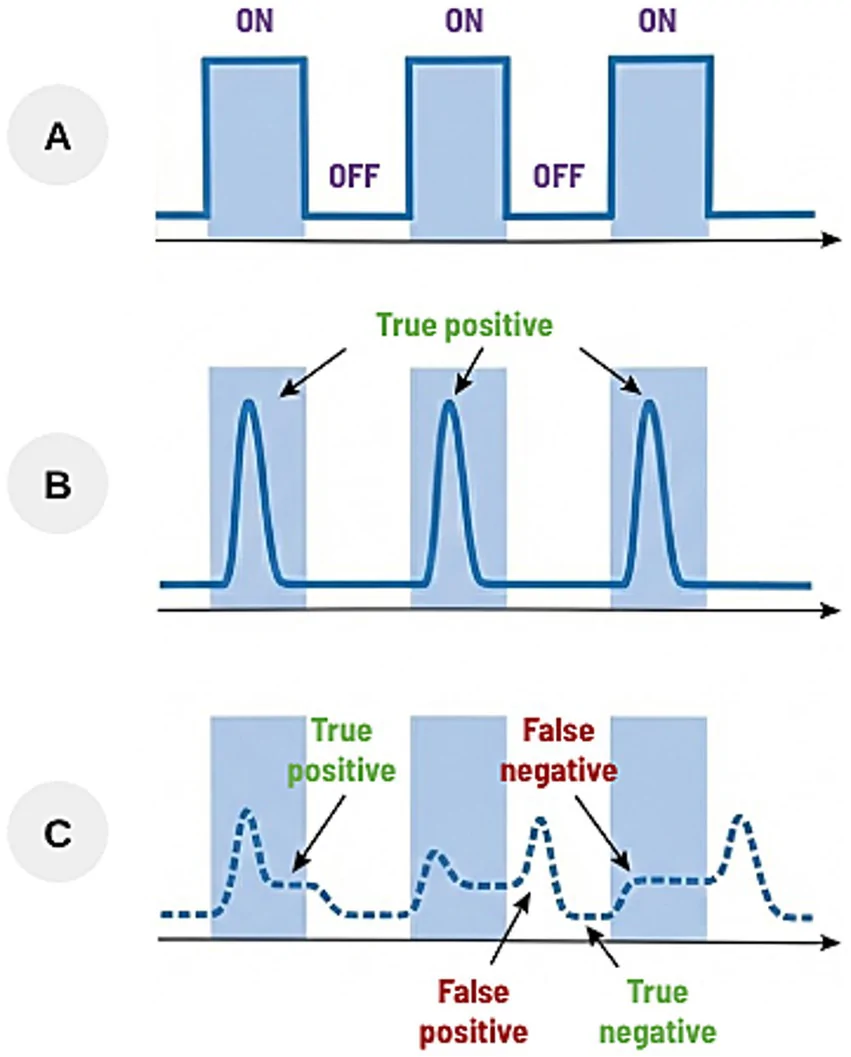
Graphical illustration of sensitivity and specificity in neurometric testing. **(A)** A controlled study design in which stimulus onset and offset are precisely regulated, constituting the “ground truth” against which a candidate metric is evaluated. **(B)** An ideal metric demonstrates clear sensitivity—a reliable response whenever the stimulus is presented (true positive)—and does not respond in its absence (true negative). **(C)** In practice, many metrics exhibit two classes of errors: false positives (a response is detected in the absence of the stimulus) and false negatives (the stimulus is present but the metric fails to respond). Achieving acceptable levels of both sensitivity and specificity constitutes a baseline requirement for any neurometric, biometric, or psychometric measure to be considered a candidate for AI training.

## 4 Stage 2: Psychometric Validation

Once a set of candidate metrics has been identified through Metric Mapping, the next critical stage is a formal assessment of each metric’s psychometric properties. This stage operationalizes and extends the four-criteria framework for valid neurometrics proposed by Ramsøy (2019): sensitivity, specificity, validity, and reliability. This is not a merely academic exercise; it is a fundamental requirement for ensuring that a metric is a trustworthy and accurate measure of the underlying psychological construct it purports to capture. Without formal validation at this stage, any AI model built upon the metric rests on an unstable and potentially invalid foundation. This is precisely the stage at which the failures documented by Varan et al. (2015) and Van Diepen et al. (2024) must be confronted and resolved.

Sensitivity is a metric’s ability to change with the presence or modulation of the target state—an emotional-engagement metric should rise consistently for an evocative advertisement relative to a neutral baseline. It is the most commonly tested criterion and the one most susceptible to effect-size inflation from the replication crisis, so validation must use stimuli representative of real commercial content, not extreme examples chosen to maximize a significant effect, and practical relevance should be judged by effect magnitude (Cohen’s *d*, partial eta-squared) rather than *p* values alone (Sullivan and Feinn, 2012). Specificity is the ability to respond selectively to the target rather than confounds—more demanding and less tested, since an EEG measure of cognitive load may also track emotional arousal. A metric lacking specificity introduces structured noise, causing a model to learn a blend of construct and confound; testing it requires designs that independently manipulate the target and plausible confounds.

Validity encompasses several distinct facets that should be kept separate. *Construct validity* asks whether the metric behaves consistently with the theory of the construct, assessed through convergent validity (correlation with established measures of the same construct) and discriminant validity (absence of correlation with unrelated constructs); the classic tool is the multitrait-multimethod matrix of Campbell and Fiske (1959), whose neglect is part of what the Varan et al. (2015) failures reflect. *Internal validity* concerns confidence that observed changes are caused by the stimulus rather than extraneous factors, requiring controls for confounds such as duration, visual complexity, and novelty. *External validity* concerns generalization to other people, settings, and times: a metric validated on university students may not hold for a diverse global population in a naturalistic digital environment.

Reliability is the criterion most directly relevant to building AI training data. Test–retest reliability assesses whether a metric yields consistent results when the same individual is measured on two occasions under equivalent conditions. The scarcity of published test–retest studies in consumer neuroscience was identified as a critical gap by Ramsøy (2019) and remains a significant deficiency, though recent work has begun to address it (Van Diepen et al., 2024). A metric with poor test–retest reliability is capturing noise rather than stable signal, and any model trained on it learns to predict random variation. Internal consistency asks whether the items, trials, or channel-level signals composing a metric measure the same construct. Parallel-form reliability—whether two operationalizations of the same construct, such as two vendors’ EEG-based measures of “engagement,” produce consistent results—is the facet most directly implicated in the findings of Varan et al. (2015). If they do not, the field has a measurement problem that no algorithmic sophistication can resolve.

Two qualifications are essential to interpreting test–retest evidence, and both have study-design implications. First, a low coefficient should not be read automatically as measurement error. Re-exposing a participant to the same stimulus does not reproduce the original measurement conditions: the second presentation is by definition a repeated exposure, and the response may legitimately differ through habituation, reduced novelty and orienting response, altered attention, or learning. The instrument may be functioning correctly and faithfully recording a genuine neural change; what has failed is the assumption that the underlying state is stable across sessions. Distinguishing true unreliability from real re-exposure effects is therefore a design problem, not merely an analytic one. Remedies include parallel stimulus forms matched on relevant dimensions rather than identical repeats, counterbalanced presentation orders, retest intervals long enough to permit decay of episodic memory for the stimulus, and explicit modeling of exposure number as a factor. Reports should always state the retest interval and whether identical or parallel stimuli were used, since the coefficient is otherwise difficult to interpret.

Second, sensitivity and reliability are distinct properties that can trade off. Experimental effects become well established precisely when between-participant variability is low, but low between-participant variability is also what depresses reliability coefficients for individual differences—the “reliability paradox” documented by Hedge et al. (2018). A paradigm with a strong record of robust group-level effects may therefore be a poor instrument for discriminating between individuals or between stimuli. This has direct consequences for Stage 3: a metric’s academic pedigree is not evidence of its suitability for AI training.

On the choice of statistic, the field should converge on the intraclass correlation coefficient (ICC) for continuous measures rather than the Pearson correlations still frequently reported. Pearson’s *r* is insensitive to systematic shifts in level between sessions: a metric whose values all drift upward on retest can yield a high correlation despite poor absolute agreement. The ICC captures both consistency and agreement, provided the appropriate form is selected and reported using the Shrout and Fleiss (1979) notation. The choice of form is not arbitrary and should follow the design and the property of interest. For the test–retest case relevant here—the same metric measured on the same participants across sessions, where what matters is whether the *same value* recurs rather than merely a consistent rank order—a two-way random-effects, absolute-agreement model is appropriate, reported as ICC(2,1) for single measurements or ICC(2,k) where the operational metric is an average of *k* measurements. A consistency model such as ICC(3,1) is appropriate only when systematic between-session shifts are not themselves a concern, which for AI training data they generally are. As a concrete baseline, we therefore recommend that test–retest reliability for candidate training metrics be reported as ICC(2,1) with its 95% confidence interval and the retest conditions stated. For interpretation, the benchmarks of Koo and Li (2016) offer a defensible starting point: below 0.50 poor, 0.50–0.75 moderate, 0.75–0.90 good, above 0.90 excellent. The confidence interval matters as much as the point estimate, since small validation samples produce intervals spanning several categories.

These benchmarks derive from clinical measurement and should not be transplanted uncritically; an acceptable coefficient depends on intended use, and a metric used to rank-order aggregated responses may tolerate lower individual-level reliability than one characterizing a single participant. As a working proposition rather than a settled threshold, metrics intended as primary training targets might reasonably be expected to show at least good reliability, ICC(2,1) ≥ 0.75, reported with a confidence interval under stated retest conditions, while those in the moderate range may remain useful as secondary features but should not carry construct claims. We use the term *AI-ready* throughout as a proposed working label for a metric that has cleared the checkpoints of this pipeline, not as an established or standardized designation; the thresholds attached to it are provisional and are offered to be tested and calibrated, not adopted as fixed criteria. Testing whether this proposition is appropriate, and how it should vary across metric classes, is itself a research priority.

Reliability also constrains the sample size at which validation studies can be considered informative, and this can be established empirically rather than assumed. Figure 2 illustrates one such approach, using Monte Carlo split-half resampling to trace how reliability estimates stabilize as sample size increases.

**Figure 2:**
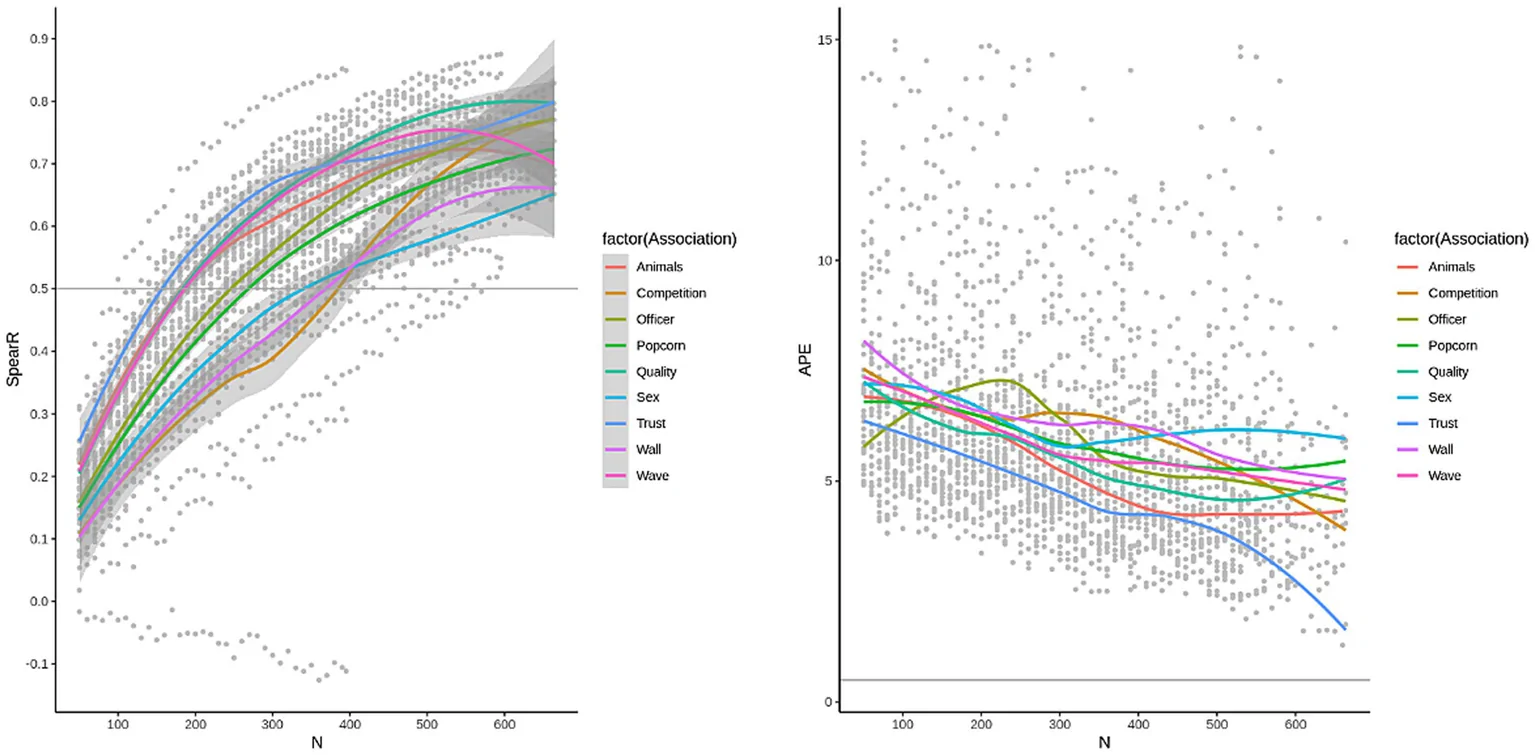
Illustration of reliability assessment methods. (Left) A Monte Carlo simulation-based split-half reliability analysis showing the effect of sample size (*n*, *x*-axis) on Spearman’s R (*y*-axis) for nine different stimulus–response associations in an implicit association test. (Right) The same associations assessed for the effect of sample size (*x*-axis) on Average Percentage Error (APE, *y*-axis) between split samples. Both panels show individual resampling draws (grey points) with a smoothed trend per association. Approaches of this kind allow the minimum sample size for a given reliability criterion to be established empirically rather than assumed: in the illustrated case, reaching a split-half Spearman’s *ρ* of 0.5 required approximately 200–400 participants depending on the association, with the most demanding associations requiring the larger figure. Establishing such thresholds per metric provides a practical, reportable benchmark for the “AI-readiness” of a measure. The data shown derive from unpublished internal analyses, are presented to illustrate the method rather than to report a general finding, and should not be generalized beyond the associations tested.

This stage of the pipeline must go beyond mere acknowledgment of these criteria and must involve formal validation studies explicitly designed to test them. The field needs to establish consensus guidelines for the sample sizes and statistical methods required for robust validation studies. Specifically, minimum sample size requirements for test–retest reliability studies with adequate statistical power, appropriate statistical tests for convergent and discriminant validity, and minimum acceptable reliability coefficients for a metric to be considered “AI-ready” must all be agreed upon. These are practical questions that need to be answered to make validation a standard, reproducible practice rather than an optional addendum.

This stage must also address the temporal dimension of validity. A metric validated at one point in time is not guaranteed to remain valid as advertising formats, cultural norms, and consumer populations evolve. This consideration foreshadows the need for ongoing model governance, which is the focus of Stage 5. The Psychometric Validation stage serves as a rigorous quality-control checkpoint, ensuring that any metric advanced to the model training stage has been formally vetted and has demonstrably earned its status as a sensitive, specific, valid, and reliable indicator of the psychological construct of interest.

## 5 Stage 3: Discriminatory Ability

A metric can successfully pass every classical psychometric test of sensitivity, specificity, validity, and reliability and still be commercially inert. This paradoxical situation arises when a metric, despite its academic credentials, fails to produce a meaningful score distribution across the actual range of stimuli that practitioners need to evaluate. This motivates the third stage in the proposed pipeline: the formal assessment of Discriminatory Ability.

Discriminatory Ability is defined here as a metric’s capacity to reliably rank-order real-world stimulus assets in a manner that maps onto meaningful differences in actual performance. It is a test of practical significance, not merely statistical significance. The underlying statistical ideas are not new. Discriminatory Ability draws directly on established concepts from clinical diagnostics and psychometrics—Receiver Operating Characteristic (ROC) analysis, Area Under the Curve (AUC), standardized effect sizes such as Cohen’s *d*, and range restriction—and it is closely connected to the reliability paradox discussed in Stage 2, since a metric with low between-stimulus variance will discriminate poorly however robust its group-level effects. What is proposed is not a novel statistical construct but the elevation of a familiar set of concerns into an explicit, named checkpoint within the pipeline.

The justification for doing so is that these concerns are routinely assumed to be satisfied in applied advertising research and are rarely tested. A metric’s psychometric credentials are typically reported; its score distribution across a representative commercial stimulus set typically is not. Making the checkpoint explicit is intended to close that gap, and to give the field a conventional place to report distributional evidence—not to claim new statistical ground.

The problem is one of range restriction and effect size in applied settings. Academic studies often use stimuli at the extremes of a continuum—a highly positive and a highly negative video—to demonstrate sensitivity, whereas advertisers evaluate multiple professionally produced versions whose differences are subtle. A metric that distinguishes a funeral from a wedding may be unable to distinguish two well-crafted commercials for the same product; their scores may differ significantly in large samples, but if the effect size is trivial the difference is meaningless for creative decisions. This pattern is prevalent in the advertising literature, where metrics show significant group differences yet distributions so compressed that most commercial stimuli are indistinguishable—a “sea of sameness” offering no signal for optimization. A model trained on such a metric is technically valid but practically inert, predicting scores with no real-world variance and offering no actionable guidance.

Assessment is therefore empirical and applied, evaluating a metric against a large, representative distribution of real-world stimuli from the relevant category—hundreds of in-market television advertisements, thousands of digital banners, or a diverse set of social video. Three questions follow. Does the metric produce a wide score distribution across the set, or are scores compressed toward one end? What is the effect size of the score difference between assets that differ on an independent in-market outcome such as sales lift or unaided recall? And does the metric produce consistent rank-ordering across random split-halves of the stimulus set, demonstrating stability of discriminatory power across independent samples?

Because the reviewers of this framework have rightly noted that the checkpoint must be actionable, the following operationalization is proposed. It is offered as a set of provisional heuristics to be calibrated against published distributional data as these accumulate, not as validated thresholds; the field does not yet possess the benchmark datasets that would fix them.

*How separation is assessed, by outcome type.* The appropriate statistic depends on the form of the outcome the metric is meant to inform. Where the outcome is binary (e.g., an advertisement passes or fails an in-market threshold), discrimination is the area under the ROC curve (AUC) relating the metric to the outcome, with an AUC near 0.5 indicating no discriminatory value. Where the outcome is continuous (e.g., sales lift), it is the strength of the metric-outcome association (e.g., Spearman’s rank correlation, which does not assume linearity) together with the standardized difference between high- and low-outcome assets. Where the outcome is itself a ranking (e.g., a preference order over creative variants), it is rank-order agreement such as Kendall’s τ or Spearman’s ρ between the metric-induced ordering and the outcome ordering. Reporting the distributional shape alongside these—the interquartile range and the proportion of the usable scale actually occupied by real stimuli—is what exposes the “sea of sameness” that a single correlation can hide.

*Normality is not required.* Because commercial score distributions are frequently skewed or multimodal, discrimination should be assessed with rank-based and distribution-free statistics rather than measures that assume normality; normality is a convenience, not a precondition, and imposing it would discard exactly the metrics whose skew is the finding of interest.

*Separating measurement error from true stimulus differences.* Apparent between-stimulus spread partly reflects measurement noise rather than genuine differences between assets. The two can be separated with a variance-components (random-effects) model that partitions total score variance into a between-stimulus component and a within-stimulus (residual) component; the ratio of between-stimulus variance to total variance is itself an intraclass correlation, and a low value indicates that observed spread is mostly noise. Equivalently, the stimulus-level reliability established in Stage 2 sets a ceiling on achievable discrimination, since a metric cannot rank stimuli more stably than it can measure them.

*Category-specific and cross-category discrimination differ and should be reported separately.* A metric may discriminate well within a homogeneous category (e.g., among skincare advertisements) yet poorly across categories, or the reverse. The relevant criterion depends on the intended deployment: a tool used to compare variants within a campaign requires within-category discrimination, whereas one used to benchmark across a portfolio requires cross-category discrimination. Both should be reported, since a metric adequate for one use may be inert for the other.

*Provisional red flags for exclusion.* Pending calibrated benchmarks, the following patterns should trigger scrutiny and, absent a compelling justification, exclusion of a metric as a primary training target: real stimuli occupying less than roughly 20% of the metric’s usable scale range; a between-stimulus variance ratio (the intraclass correlation above) below about 0.5, indicating that most observed spread is noise; strong skew combined with a floor or ceiling effect, such that a large majority of stimuli fall in one or two bins; and split-half rank-order agreement below about 0.5, indicating that the metric’s ordering of stimuli does not replicate. These figures are deliberately round and provisional; their purpose is to make the checkpoint usable now while the distributional evidence needed to refine them is gathered and published.

The practical consequence of applying this test is that a metric may be excluded from an AI training pipeline on grounds of discriminatory failure rather than psychometric failure—a distinction for which the field currently has neither a vocabulary nor a conventional place to report. A metric that performs well in traditional validation studies may nonetheless produce score distributions across real advertising stimuli so compressed that observed differences between assets are practically meaningless for creative optimization, and such a metric should not enter a training corpus regardless of its psychometric credentials.

Publishing score distributions across representative commercial stimulus sets, alongside conventional validation statistics, would allow this judgment to be made transparently and would supply the distributional benchmarks the field presently lacks. By formalizing Discriminatory Ability as an independent stage, metrics used to train AI models can be assessed not only for academic soundness but also for practical informativeness.

## 6 Stage 4: Principled Data Scaling and Model Development

With a set of metrics that have been mapped, validated, and confirmed for Discriminatory Ability, the pipeline advances to Stage 4: the large-scale, principled data collection and model development required to build a robust predictive system. Metric validity is a necessary but not a sufficient condition for a sound AI system. A model trained on impeccably validated metrics can still be worthless if it is fitted, validated, or reported badly, and generalizability is as much a property of model design and evaluation as it is of data quality. Five elements are therefore essential at this stage: the scale and diversity of the training data, the representativeness of that data across populations, the explicit definition of outcome anchoring, the soundness of the model development and validation procedure, and the adoption of new norms of transparency in model disclosure.

### 6.1 Scale and diversity of training data

The performance and generalizability of any machine learning model are critically dependent on the scale and diversity of the data it learns from. Consumer neuroscience has, for practical and financial reasons, historically relied on studies with small sample sizes—typically tens of participants and a handful of stimuli. While the neuroforecasting literature has shown that neural responses from a small group can be surprisingly predictive of population-level outcomes, building a generalizable AI model for advertising effectiveness requires a dataset of fundamentally different magnitude. The training set must be large enough to learn stable, non-spurious patterns, which in practice means thousands of evaluated stimuli, not dozens. A model trained on a small, homogeneous set of advertisements will overfit to the idiosyncratic features of that set and will fail to generalize to new, unseen creative content.

Equally important is systematic diversity. Advertising spans product categories, cultural contexts, media formats (television, digital video, social media, out-of-home, audio), and creative strategies (humor, emotion, information, social proof, celebrity endorsement). A model trained predominantly on thirty-second television commercials for fast-moving consumer goods will perform poorly on a six-second mobile video for a luxury brand. Training data must therefore be curated to represent the actual variation space of real-world advertising, which requires sustained investment in acquisition and annotation and a systematic approach to avoiding bias toward any particular category, format, or cultural context.

### 6.2 Representativeness and bias in training data

Diversity of content must be accompanied by representativeness of people. A corpus can span every media format and product category while still encoding a narrow view of who consumers are. Consumer neuroscience inherits the sampling problems of psychology more broadly, in which convenience samples drawn from wealthy, educated, industrialized populations have long been treated as representative of humanity in general (Henrich et al., 2010). Further skew is specific to biometric measurement: automated facial coding has been shown to perform unequally across demographic groups (Buolamwini and Gebru, 2018); EEG signal quality varies with hair texture and electrode fit, and standard electrodes can yield markedly higher impedance on coarse and tightly curled hair, degrading data quality for participants of African descent unless the hardware is explicitly adapted (Etienne et al., 2020); and optical sensors such as those used in photoplethysmography are systematically affected by skin pigmentation, a bias documented in detail for pulse oximetry (Sjoding et al., 2020) and applicable to the optical cardiac and electrodermal sensing used in consumer biometrics.

A model trained predominantly on one demographic profile may generate confident but systematically inaccurate predictions for audiences unlike it, and because the outputs are numerical and automated, such errors are easily mistaken for objectivity. Three requirements follow: training corpora should be audited for demographic and cultural composition; performance should be reported disaggregated by subgroup rather than as a single pooled figure; and known gaps in coverage should be disclosed. This matters with particular force if the framework is to inform evaluation of systems deployed across diverse markets.

### 6.3 Outcome anchoring

The model’s outcome anchoring must be explicitly defined and understood. An AI model is trained to predict a specific outcome variable—the “ground truth”—and the choice of this variable is a critical decision that determines what the model is actually predicting. In consumer neuroscience, there is a fundamental distinction between models trained on laboratory-based behavioral proxies and those trained on in-market outcomes. Laboratory proxies—such as ad recall, brand recognition, or forced-choice preference—are easier and less expensive to collect but may not be perfect correlates of real-world consumer behavior. In-market outcomes—such as sales lift, click-through rates, or changes in brand tracking metrics—are the ultimate measure of commercial success, but are much harder to obtain and subject to many confounding factors.

Neither approach is inherently superior, but the distinction must be transparent. A model trained to predict laboratory-assessed ad recall is not the same instrument as one trained to predict sales lift, and users of the model must clearly understand what it predicts in order to interpret its outputs appropriately and use it for the right decisions. The failure to communicate this distinction transparently is a form of scientific misrepresentation that undermines trust in the field.

### 6.4 Model development and validation

The validation effort invested in Stages 1 through 3 can be undone by careless model development. Four requirements deserve explicit statement, because they are frequently absent from both published and commercial claims in this domain.

*Model family and assumption checking*. Model family should be justified rather than defaulted to. Neurophysiological training sets here are typically wide and short—many derived features, comparatively few independent stimuli—precisely the regime in which high-capacity models overfit. Where a regularized linear model or modest tree ensemble performs comparably to a deep architecture, the simpler model is preferable on grounds of interpretability, stability, and auditability. Assumptions should be checked rather than presumed: distributional properties of derived metrics, non-independence arising from repeated measurement within participants and stimuli, and heteroscedasticity across stimulus types all bear on whether a model and its uncertainty estimates are appropriate.

*Validation strategy and data leakage*. Standard random k-fold cross-validation is frequently inappropriate here and will produce optimistic estimates. Observations from the same participant, session, stimulus, or campaign are not independent, and splitting them randomly across folds allows information to leak between training and test sets—a documented and widespread cause of irreproducible findings in machine-learning-based science (Kapoor and Narayanan, 2023). Partitioning should instead respect data structure: grouped or blocked cross-validation holding out participants and stimuli in their entirety, and time-series or forward-chaining designs where temporal ordering matters. Preprocessing that learns parameters from data—normalization, feature selection, artifact-rejection thresholds—must be fitted inside the training fold.

*Uncertainty*. Point estimates of accuracy are insufficient. Cross-validated estimates carry considerably wider error bars than is commonly appreciated at typical neurophysiological sample sizes, and the standard error across folds substantially underestimates true variability (Varoquaux, 2018). Performance should be reported with confidence intervals and benchmarked against a null model and a simple baseline, so readers can judge whether an improvement is distinguishable from noise (Poldrack et al., 2020).

*External validation and the generalization claim*. Internal cross-validation establishes only that a model generalizes within the distribution its training data were drawn from. A claim that a system predicts advertising performance requires evaluation on a genuinely held-out set, and—crucially—the axis along which data are held out must match the generalization the system claims to support. “Held out” is not a single thing; it names a family of increasingly demanding tests, each licensing a different claim:

- *Participant holdout*—new people, same stimuli—supports a claim of generalization to new audiences but not to new creative.
- *Stimulus holdout*—new advertisements, same participants—supports generalization to new creative within the sampled population.
- *Brand or campaign holdout*—no asset from a held-out brand or campaign appears in training—guards against learning brand-specific rather than general features.
- *Category holdout*—an entire product category withheld—tests whether the model transfers across categories, the claim implied whenever a tool is sold as category-agnostic.
- *Country or market holdout*—a different market or culture—tests the geographic generalization most cross-border advertisers assume.
- *Temporal holdout*—evaluation on a later period than training—tests robustness to the drift discussed in Stage 5 and is the closest offline proxy for real deployment.
- *Device or laboratory holdout*—a different acquisition system or site—tests whether the model has learned psychology or hardware signatures, the concern raised in Section 2.3.

A system should be validated on whichever of these corresponds to its intended use, and its efficacy claims should be scoped to what was actually tested: a model validated only on participant holdout has no evidential basis for a claim of cross-category or cross-market prediction. Reporting external alongside internal performance should be a minimum expectation for any efficacy claim, and the degradation observed across each holdout axis is itself informative about what the model has genuinely learned.

Validation of this kind operates at two complementary levels. Figure 3 illustrates the quantitative level, aggregating agreement between predicted and measured responses across large stimulus sets and disaggregating it by category; Figure 4 illustrates the qualitative level, comparing measured and model-predicted attention on individual stimuli. Neither is sufficient alone: aggregate coefficients can conceal category-specific failure, while qualitative comparison is intuitive but unsystematic.

**Figure 3:**
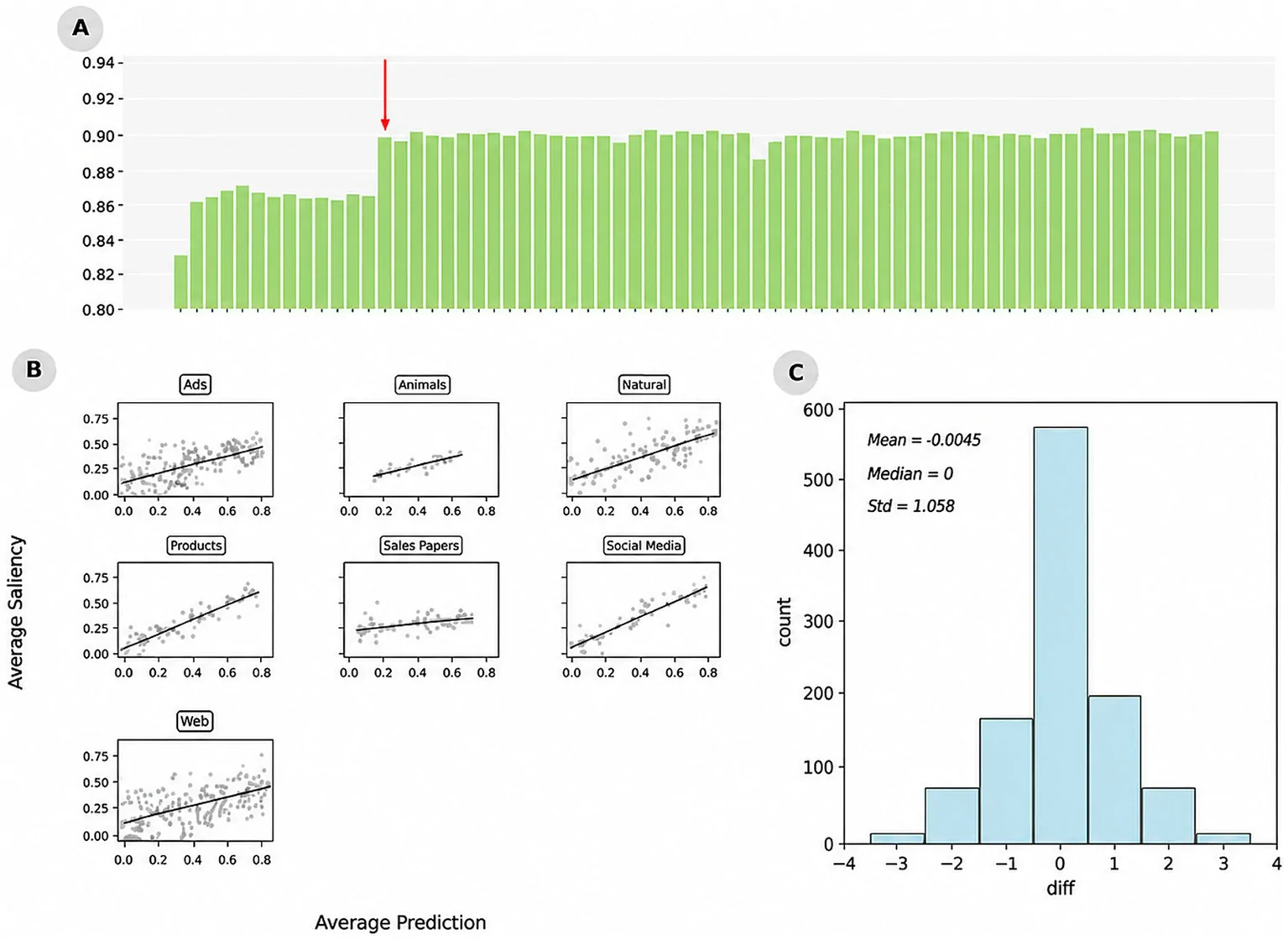
Illustrative approaches to the quantitative assessment of model accuracy, using eye-tracking-based attention prediction as an example. **(A)** Pixel-by-pixel agreement between predicted and measured attention heat maps, aggregated into a single accuracy coefficient. Each bar represents one model architecture in development order; the red arrow marks a step change in accuracy associated with an architectural change. **(B)** The same analysis disaggregated by stimulus category (advertising, animals, natural images, product images, sales papers, social media, and web content), enabling category-specific optimization; for neuromarketing applications, accuracy on advertising content is the relevant benchmark. An equivalent breakdown at the area-of-interest level (brand, product, headline, body copy, call to action) permits further refinement. **(C)** Distribution of rating differences from a double-blind design in which participants, blinded to whether a displayed heat map was model-generated or measured, rated attention to specified advertisement elements. A difference distribution centered near zero is not by itself evidence of equivalence: formal equivalence testing against a pre-specified margin, such as a two one-sided tests procedure, would be required to support such a conclusion, and is recommended as standard practice for validation of this kind. The panels are presented to illustrate the forms such assessment can take. They derive from unpublished internal analyses conducted as commercial research, are shown for illustrative purposes only, and are not offered as generalizable empirical findings.

**Figure 4:**
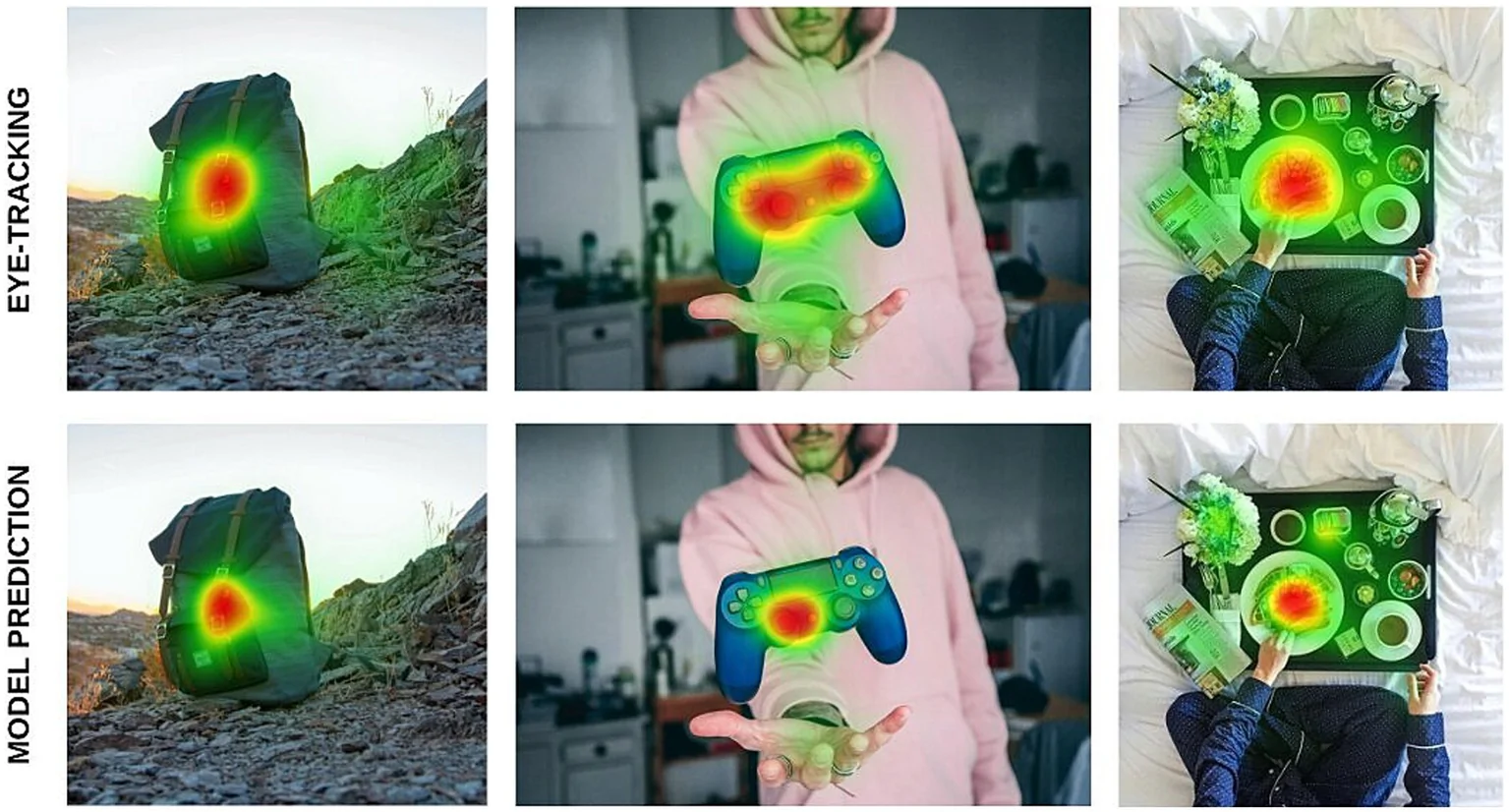
Qualitative comparison of measured and model-predicted visual attention. Each column shows a single static image stimulus, with the aggregated eye-tracking heat map above and the corresponding model prediction below; warmer colors indicate greater measured or predicted attention. All three stimuli were evaluated with an image-based model and span different scene types: an isolated product in a natural setting, a product held by a person, and a multi-element overhead composition. Comparisons of this kind provide an interpretive check on model behavior—whether the model attends to the same regions as a human sample, and where the two diverge—but they are illustrative only and cannot substitute for the quantitative assessment shown in Figure 3. The stimuli are internal materials and the heat maps derive from unpublished internal analyses, presented for illustration and not as generalizable empirical findings.

### 6.5 Interpretability and explanation

Valid prediction is not the only objective. Understanding *why* a model produces a given prediction matters for responsible deployment, with particular force here because outputs are routinely described in psychological terms. When a system reports that an advertisement scores highly on “attention” or “memory encoding,” it invites a mechanistic reading that a purely predictive model may not support. Techniques from explainable AI—feature attribution, saliency methods, counterfactual analysis, partial dependence—can help establish whether predictions rest on features consistent with the rationale established in Stage 1, or on incidental correlates of the training set such as production style, brand familiarity, or format convention (Doshi-Velez and Kim, 2017).

Two cautions apply. Post-hoc explanations approximate a model’s behavior and can themselves be unstable or misleading, an argument for preferring inherently interpretable models where performance permits (Rudin, 2019). And interpretability is not a substitute for validity: showing that a model relies on a given feature says nothing about whether that feature measures the construct it is named after. The two are complementary, and a system making construct claims requires both.

### 6.6 Transparency in model disclosure

Finally, the field must establish new norms for transparency in the disclosure of commercial AI models. While proprietary algorithms and specific model weightings will and should remain protected as intellectual property, the current “black box” approach to commercial neuromarketing AI is unsustainable if the field is to maintain scientific credibility. At a minimum, the general class of machine learning approach used (e.g., gradient-boosted trees, convolutional neural network, vision transformer), the input metrics, the outcome variable on which the model was trained, and the external validation performance should be disclosed when efficacy claims are made. This minimum standard of disclosure is what is required for an independent researcher or sophisticated client to assess the plausibility of a model’s claims.

The analogy to the pharmaceutical industry is instructive: a pharmaceutical company must disclose a drug’s pharmacological mechanism of action even while the specific synthesis process remains a trade secret. This level of transparency does not threaten commercial viability; it is a prerequisite for scientific credibility and the long-term trust on which the industry’s commercial model depends.

## 7 Stage 5: Model Governance and Drift Prevention

The final stage addresses a well-documented challenge in the machine learning literature that has received almost no formal treatment in consumer neuroscience: model drift. Drift—also termed concept or data drift—is the degradation of predictive performance over time as the statistical properties of the data a model operates on change (Gama et al., 2014; Kustitskaya et al., 2025). A model is trained on a snapshot of the world; when consumer behavior evolves, creative norms shift, or new platforms emerge, accuracy can decay rapidly and silently. For a field aiming to predict behavior that is inherently dynamic and context-dependent, the absence of a formal governance framework is a consequential gap.

The drivers of drift in this domain are numerous and specific. Creative norms evolve continuously: what counts as visually salient or emotionally engaging changes with platform design (the rise of short-form vertical video on TikTok and Instagram Reels), cultural trends, and audience expectation, and a model trained on the visual grammar of 2018 television advertising will have learned features—pacing conventions, aspect ratios, narrative structures—increasingly irrelevant to the mobile-first content that now dominates. Media consumption habits shift in parallel: attention to a six-second, sound-off mobile advertisement differs fundamentally from attention to a thirty-second commercial in a lean-back environment, the biometric responses to the two are likely qualitatively different, and a model trained on one cannot be assumed to generalize to the other. The demographic and psychographic composition of consumer panels and populations also shifts over time, as does the cultural context in which advertising is received, and post-pandemic changes in media routines illustrate how rapidly such shifts can occur.

A further and rapidly growing driver is the generative production of creative assets itself. As advertising content is increasingly generated or heavily assisted by generative AI systems, the statistical properties of the stimulus distribution change—in visual style, pacing, compositional convention, and the sheer volume of variants produced—and they change on a timescale far shorter than traditional creative cycles. A predictive model trained on human-produced creative may encounter generated content that lies outside its training distribution. The feedback loop is also potentially self-reinforcing: if generated variants are selected on the basis of model predictions, subsequent training data will be enriched for assets the model already favors, narrowing the effective distribution over time. This is a strong argument for treating drift monitoring as a standing requirement rather than a periodic audit.

The COVID-19 pandemic provided a dramatic example across many applied AI domains, as sudden disruptions to consumer behavior degraded deployed models in applications from demand forecasting to fraud detection. Such episodes are an acute instance of a chronic condition: deployed systems accumulate maintenance obligations that are easy to defer and costly to ignore (Sculley et al., 2015), and the operational difficulty of detecting and correcting post-deployment decay is well documented (Paleyes et al., 2022). Analogous if more gradual shifts occur continuously in the consumer landscape, and models that are not actively managed will become less accurate over time—a predictable consequence of deploying any machine learning system in a non-stationary environment.

Three governance practices, drawn from established practice in other applied machine learning domains such as financial services and healthcare AI, should become standard requirements for any commercially deployed neuromarketing AI system. They are analogous to post-market surveillance, a regulatory requirement for medical devices and pharmaceuticals (U.S. Food and Drug Administration, 2024).

Retraining is the most fundamental governance practice. Models should be retrained on fresh data that has itself passed through Stages 1–3; feeding raw, unvalidated data into a retraining cycle would defeat the pipeline’s purpose. A fixed calendar interval is the simplest trigger but is hard to justify on evidence and tends to be either wasteful or too slow. Where a defensible interval cannot be established, retraining should instead be triggered by measurable conditions: a drift trigger when input or prediction distributions move beyond a preset bound (below); a performance trigger when calibration against incoming ground truth falls below a threshold; or an event trigger when a known shift occurs, such as a platform introducing a new dominant format. Whichever governs, retraining should be proactive rather than a reaction to degradation already acted upon in consequential decisions.

Drift Detection is the second practice. The statistical properties of a model’s inputs and outputs should be monitored continuously, analogously to statistical process control in manufacturing, so that deviations from the expected operating range are caught early. Alert thresholds can flag when the distribution of input metric scores or of prediction scores shifts inconsistently with expected natural variation, allowing investigation and targeted retraining before performance degrades materially. Established methods include population stability indices, Kullback–Leibler divergence, and model-based detectors (Gama et al., 2014; Kustitskaya et al., 2025).

Ground Truth Feedback Loops constitute the third and most difficult governance practice. Predictions should be periodically calibrated against real in-market outcomes, but feedback loops in advertising are long and noisy: months may separate testing from measurable sales impact, and competitor activity, pricing, distribution changes, and seasonality all intervene. Formalizing the link between predictions and outcomes is nonetheless what allows real-world performance to be understood and calibrated over time; without it, the relationship between neurophysiological prediction and commercial outcome cannot be established, and models cannot be verifiably improved.

For governance to be auditable rather than aspirational, these practices should attach to specific, measurable indicators. At minimum, a governed system should monitor: input drift (movement in the distribution of the metric scores fed to the model) and concept drift (change in the relationship between those inputs and the outcome), quantified with the detectors named above; calibration drift (divergence between predicted and observed outcomes as ground truth arrives); and subgroup performance (accuracy tracked separately across the demographic, category, and format subgroups identified in Section 6.2, so that aggregate stability does not mask localized failure). Supporting this monitoring requires disciplined data and model versioning—a recorded lineage of which data trained which model version, so that a regression can be traced and reversed—together with predefined retraining triggers as above, independent audit conditions specifying when and by whom a deployed model is reviewed, and explicit model-retirement criteria stating the performance or drift level at which a model is withdrawn from service rather than patched. Making these indicators explicit is what would allow the certification or auditing mechanisms discussed in the Conclusion to be applied in practice.

By introducing Model Governance and Drift Prevention as a formal, named stage of the pipeline, the final stage transforms the process of building an AI model from a discrete one-time event into a continuous, managed lifecycle. It acknowledges that in a dynamic world, predictive accuracy is not a static property but an active state that must be maintained through sustained investment in data collection, monitoring, retraining, and calibration.

## 8 The pipeline as a decision structure

The five stages are summarized in Table 1 as an explicit decision structure. For each stage it states the evidence a metric or model must provide, the recommended additional evidence, the relevant statistical procedures, the indicators that signal failure, the corrective action that failure implies, and the condition that must be met before proceeding. The numerical values that appear are the provisional working proposals introduced in the preceding sections; where no evidence-based threshold yet exists, this is stated rather than concealed. The table is intended as a practical checklist, not as a validated standard, and the thresholds are offered to be calibrated as distributional and reliability data accumulate.

**Table 1:** The five-stage pipeline as a decision structure. Thresholds shown are provisional working proposals, not validated standards.

Stage	Required evidence	Recommended evidence	Statistical procedures	Failure indicators	Corrective action	Condition to proceed
1. Metric Mapping	Documented mechanistic rationale linking metric to target behavior; auditable metric map	Structured, PRISMA-style search; human-verified search for disconfirming evidence	Evidence appraisal; quality rating of sources	No mechanistic rationale; support rests on a single unreplicated study; evidence surfaced only by unaudited automated search	Seek primary and replication evidence; deliberate search for null results; drop metric if rationale absent	A coherent, testable rationale and an appraised evidence base are documented
2. Psychometric Validation	Sensitivity, specificity, and reliability evidence under representative stimuli	Convergent/discriminant validity; parallel-form checks across methods	ICC(2,1) with 95% CI for test–retest; effect sizes with CIs for sensitivity; MTMM for construct validity	ICC(2,1) < 0.75 for a primary target; poor specificity; parallel forms uncorrelated	Redesign with parallel stimuli and adequate power; demote to secondary feature; exclude if unreliable	Metric meets the reliability proposition under stated retest conditions
3. Discriminatory Ability	Score distribution and metric-outcome separation across a representative commercial stimulus set	Within- and cross-category discrimination reported separately; split-half stability	AUC (binary), rank correlation and standardized difference (continuous), Kendall’s τ (ranked); variance-components model to separate true from noise variance	Real stimuli occupy <~20% of usable range; between-stimulus variance ratio <~0.5; split-half rank agreement <~0.5; floor/ceiling with strong skew	Exclude as primary training target; retain only as secondary feature if justified	Metric separates real stimuli in a way that maps onto a meaningful outcome
4. Data Scaling and Model Development	Large, diverse, demographically audited training data; leakage-controlled validation; external validation matched to the generalization claim	Subgroup-disaggregated performance; interpretability check; disclosure of model class and outcome	Grouped/blocked and forward-chaining CV; variance-components for uncertainty; external-set evaluation; performance with CIs vs. null and baseline	Random k-fold used; leakage across participants/stimuli/campaigns; no external validation; large internal-to-external degradation	Re-partition to respect data structure; collect external set; re-scope efficacy claim to what was tested	Model generalizes on the held-out axis matching its intended use
5. Model Governance and Drift	Monitoring of input, concept, and calibration drift; subgroup tracking; versioning; defined retraining triggers	Independent audit conditions; ground-truth feedback loop; model-retirement criteria	Population stability index, KL divergence, model-based drift detectors; calibration tracking	Drift beyond preset bounds; calibration decay; localized subgroup failure masked by aggregate stability	Trigger retraining on re-validated data; investigate subgroup failure; retire model if criteria met	Monitoring and retraining infrastructure is in place and active

## 9 A structured hypothetical application

To illustrate how the pipeline shapes practical decisions, consider a hypothetical evaluation of frontal alpha asymmetry (FAA) as a candidate metric for predicting approach motivation toward video advertising. The example is constructed for expository purposes and is not a report of empirical results; the values discussed are illustrative, and no claim is made that they describe the actual properties of this metric. Its purpose is to show where a framework of this kind changes a decision that would otherwise be made implicitly.

*Stage 1 (Metric Mapping)*. FAA has an explicit theoretical rationale: relative left-frontal activation has been associated with approach motivation, providing a mechanistic chain from signal to construct to the consumer behavior of interest. A structured review would document this rationale alongside known confounds—sensitivity to reference scheme, individual differences in resting asymmetry, and the contested status of alpha power as an inverse index of cortical activation—and would record the practical constraints of frontal electrode placement in naturalistic settings. FAA passes this stage, but with documented caveats rather than unqualified endorsement.

*Stage 2 (Psychometric Validation)*. The metric would be assessed for sensitivity to approach-motivating content, specificity against arousal and cognitive load, and reliability. Suppose test–retest assessment using identical stimulus repeats returns an ICC in the moderate range. Under the framework, this result would not be interpreted immediately as measurement failure: repeated exposure to the same advertisement plausibly reduces novelty and orienting response, so a follow-up using parallel matched stimuli and a longer retest interval would be required to separate genuine instability from re-exposure effects. The decision rule is that the metric cannot proceed as a primary training target until this ambiguity is resolved, with the specific ICC form and its confidence interval reported.

*Stage 3 (Discriminatory Ability)*. FAA would then be scored across a large, representative set of in-market advertisements rather than extreme exemplars. The critical question is distributional: if scores cluster tightly, so that most professionally produced advertisements are indistinguishable, the metric fails this checkpoint irrespective of its Stage 2 performance. This is the point at which a metric with strong academic credentials may be excluded—a judgment for which current practice provides no conventional place to make or report.

*Stage 4 (Data Scaling and Model Development)*. If FAA proceeds, training data would be collected across formats, categories, and demographically audited samples, with the outcome variable stated explicitly. Cross-validation would hold out participants and stimuli as whole groups, preprocessing parameters would be fitted within training folds only, and performance would be reported with confidence intervals and validated externally on unseen campaigns.

*Stage 5 (Governance)*. Deployment would trigger monitoring of the input and prediction distributions, a defined retraining schedule, and periodic calibration against available in-market outcomes.

The example illustrates the framework’s principal practical consequence: a metric can fail at Stage 3 having passed Stage 2, and the two failures call for different responses. Without an explicit checkpoint, that distinction is easily missed.

## 10 Discussion

The five-stage pipeline proposed in this article is best understood as an argument for shifting the balance of attention in consumer neuroscience away from algorithmic sophistication and toward measurement quality and methodological transparency. Its primary contribution is scholarly: it defines a structured research agenda, identifies where the field’s evidence base is thinnest, and specifies what would count as progress at each stage. It also carries implications for applied practice, which are discussed below as a secondary consequence rather than as the paper’s purpose.

### 10.1 Implications for academic research

For academic researchers, each stage raises open empirical questions. How should the strength of theoretical grounding for emerging signals be operationalized? What reliability and effect-size benchmarks suit different classes of neurometric, and what minimum coefficient qualifies a metric as “AI-ready”? What distributional properties should a metric show to count as discriminating, and what benchmark datasets would allow comparison across metrics and vendors? How can AI-derived perceptual metrics be validated when they lack a physiological basis? What drives drift in different contexts, and what retraining triggers follow? These require collaborative, longitudinal research programs rather than isolated studies.

### 10.2 Implications for tool developers

For those developing applied tools, the framework suggests a way through the tension between commercial confidentiality and scientific transparency. Transparency of validation evidence is not equivalent to transparency of algorithmic implementation. An organization can protect its proprietary model architecture and weightings while still reporting that its input metrics have been validated according to the criteria of Stages 1 through 3, that its models have been externally validated as described in Stage 4, and that deployment is governed by the practices of Stage 5.

Comparable arrangements operate in other regulated domains, where mechanism and evidence of efficacy are disclosed while manufacturing detail remains protected. Whether such norms can be established in this field is an open question, and it is likely to depend more on collective agreement among researchers, practitioners, and journals than on any framework advanced by a single author.

### 10.3 Implications for the evaluation of applied systems

A secondary implication concerns those who evaluate applied predictive systems, whether as reviewers, collaborators, or purchasers. Existing industry guidance, such as that issued by ESOMAR and the Advertising Research Foundation (ARF), offers valuable ethical and practical advice but does not supply a technical framework for assessing the validity of AI-based predictive tools.

The stages outlined here suggest the categories of evidence such an assessment would require: the theoretical rationale for the chosen input metrics; reliability evidence with the statistic, its confidence interval, and the retest conditions specified; evidence of Discriminatory Ability on a representative commercial stimulus set; a description of the training data, its demographic composition, and its outcome anchoring; evidence of external validation on genuinely held-out data; and a stated approach to monitoring, drift detection, and retraining. These are the same categories that would structure an academic evaluation of such a system. The point is not to prescribe a commercial process but to observe that the scientific and applied evaluation criteria coincide, and that neither is currently met as a matter of routine.

### 10.4 Limitations of the proposed framework

This article presents a conceptual framework, and several limitations should be stated plainly.

First, the pipeline has not been empirically evaluated. No study has compared model performance or robustness under validation-centered versus validation-naïve development in this domain, and until such comparisons exist, the framework’s central claim—that this sequence of checkpoints materially improves outcomes—remains a reasoned proposition rather than a demonstrated result.

Second, the components of the pipeline are not individually novel. Metric screening, Psychometric Validation, data quality control, outcome anchoring, and lifecycle governance are established concerns in measurement science and applied machine learning. The contribution is integrative: assembling them into a domain-specific sequence and identifying where consumer neuroscience currently omits them. Discriminatory Ability in particular is a repositioning of familiar ideas about effect size and range restriction rather than a new statistical construct.

Third, the operational guidance offered here is partial. The pipeline is necessarily general, and its application must be adapted to different metrics, media formats, and organizational contexts. The reliability benchmark suggested in Stage 2 is adapted from clinical measurement and may not transfer cleanly; no comparable numerical benchmark is proposed for Discriminatory Ability, because the distributional data required to establish one have not been published; and retraining intervals are discussed qualitatively because the empirical basis for specifying them does not yet exist. These gaps are consequences of the field’s current evidence base, and identifying them precisely is part of the paper’s intended contribution, but they mean the framework is better described as a scaffold for future standardization than as a standard.

Fourth, the framework addresses measurement quality but does not, in isolation, resolve all sources of model invalidity. Even with well-validated metrics, generalizability can be limited by unrepresentative training data, inappropriate model architectures, or the inherent unpredictability of consumer behavior in novel contexts.

Fifth, the perspective advanced here is shaped by the author’s position in the field, including commercial involvement in applied consumer neuroscience. The arguments are offered on their merits, and the framework is intended to apply to the author’s own organization as much as to any other, but readers should weigh the disclosure provided below.

### 10.5 The cultural shift required

Adopting a framework of this kind would require sustained investment in the foundational work of metric validation—less visible than building novel architectures, but arguably more consequential for the field’s long-term credibility. It would also require a commitment to ongoing model governance, demanding resources and institutional discipline that are easy to deprioritize in competitive environments, and a willingness to move away from the “black box” as a selling point toward a more transparent conversation about what consumer neuroscience AI can and cannot reliably do. Such transparency may feel like a competitive vulnerability in the short term, but it is the foundation on which a scientifically credible field must be built.

## 11 Conclusion

The promise of artificial intelligence in consumer neuroscience is real. Predicting consumer responses from media assets alone, without the time and cost of primary data collection, would genuinely transform how advertising is created, tested, and optimized, and the three-stage progression from individual neural prediction to neuroforecasting to AI-driven prediction has brought the field to that threshold.

But crossing this threshold responsibly is likely to require foundational work that precedes the algorithm. The replication crisis, the parallel-form validity failures documented by Varan et al. (2015) and Van Diepen et al. (2024), and the inherent complexity of biostatistical data are unlikely to be resolved by more sophisticated modeling alone. These are, in the argument advanced here, problems that should be addressed at the level of measurement before they can be reliably addressed at the level of prediction—a claim that itself requires empirical evaluation. A predictive model can be no more valid than the constructs it is trained to predict.

The five-stage pipeline proposed here—Metric Mapping, Psychometric Validation, Discriminatory Ability, Principled Data Scaling and Model Development, and Model Governance and Drift Prevention—offers one structured way of organizing this foundational work. It is presented as a conceptual synthesis and a research agenda rather than a validated standard, and its value will depend on whether the empirical work it identifies is actually undertaken.

One direction deserves particular attention. If the stages described here can be operationalized with sufficient precision, they could form the basis for independent certification or algorithmic auditing of applied predictive systems. Analogous mechanisms already exist in other domains where automated systems inform consequential decisions, and the documentation practices now emerging around AI governance point in the same direction. A verifiable third-party assessment of whether a system’s input metrics have been validated, whether its models have been externally tested, and whether its performance is actively monitored would allow claims to be evaluated without requiring disclosure of proprietary algorithms. Developing the benchmarks and procedures such a mechanism would require is a substantial undertaking, and it is not attempted here; but it is a natural extension of this framework, and a more productive destination for the argument than continued advocacy.

A predictive model is, like any scientific instrument, only as good as the care with which it is built, validated, and maintained. Whether consumer neuroscience realizes the potential of AI to understand and predict human behavior may depend considerably on whether the field invests in the validation infrastructure that this potential appears to require—a proposition this paper advances as a research agenda rather than a settled conclusion.

## Data Availability

The data analyzed in this study is subject to the following licenses/restrictions: The illustrative data underlying Figures 2–4 are the proprietary property of Neurons and cannot be made publicly available, as they form part of the company’s commercial research assets; this restriction is a condition of their commercial origin rather than a choice specific to this article. No inferential claims rest on these data, and the figures are illustrative only. No other new data were generated or analyzed in this study, and all sources supporting the arguments made are cited in the reference list. Requests regarding the illustrative material may be directed to the author. Requests to access these datasets should be directed to TR, Neurons, Taastrup, Denmark. Email: thomas@neuronsinc.com.
